# New Clinical Markers of Oxidized Lipid-Associated Protein Damage in Children and Adolescents with Obesity

**DOI:** 10.3390/children11030314

**Published:** 2024-03-06

**Authors:** Eirini Kostopoulou, Athina Varemmenou, Electra Kalaitzopoulou, Polyxeni Papadea, Marianna Skipitari, Andrea Paola Rojas Gil, Bessie E. Spiliotis, Sotirios Fouzas, Christos D. Georgiou

**Affiliations:** 1 Department of Pediatrics, School of Medicine, University of Patras, 26500 Patras, Greece; irekost@upatras.gr (E.K.); spilioti@upatras.gr (B.E.S.); 2 Department of Medicine, University of Patras, 26504 Patras, Greece; up1048042@ac.upatras.gr; 3 Department of Biology, University of Patras, 26504 Patras, Greece; ilektra.kalaitzopoulou@upatras.gr (E.K.); bio3669@ac.upatras.gr (P.P.); marianna.skipitari@ac.upatras.gr (M.S.); c.georgiou@upatras.gr (C.D.G.); 4 Laboratory of Basic Health Sciences, Department of Nursing, Faculty of Health Sciences, University of Peloponnese, 22100 Tripoli, Greece; apaola71@yahoo.com.mx

**Keywords:** PrMDA, PrTBARS, LOOH, oxidative stress, obesity, children

## Abstract

Obesity in children and adolescents has been associated with oxidative stress (OS). The lipid hydroperoxides (LOOH) and the malondialdehyde (MDA) and thiobarbituric reactive substances (TBARS) that oxidatively modify proteins (Pr) (i.e., PrMDA and PrTBARS, respectively) represent markers of OS-associated lipid peroxidation. We aimed to assess OS in children and adolescents with obesity using—for the first time—markers involved in the early and late lipid oxidation process. LOOH, PrMDA, and PrTBARS were investigated in 41 children and adolescents with obesity and 31 controls. Obesity was defined as BMI > 95% for age and sex. The PrMDA/PrTBARS pair, which reflects a late peroxidation stage, was found to be significantly high (39%/180%) in children and adolescents with obesity compared to controls (*p* < 0.001). Similarly, the early LOOH peroxidation stage marker was increased by 30%. The studied OS parameters were not influenced by sex or age. Our study introduces LOOH, PrTBARS, and PrMDA as markers for evaluating OS in children and adolescents with obesity. LOOH, PrTBARS, and PrMDA may also hold promise as prognostic markers for potential obesity-associated long-term complications.

## 1. Introduction

Obesity has been associated with oxidative stress (OS) in adults; a positive association has been observed between indices of obesity, such as body mass index (BMI) and waist/hip ratio, and urinary levels of 8-epi-prostaglandin F2alpha (8-epi-PGF2). 8-epi-PGF2 constitutes a major F2 isoprostane produced in vivo by free radical-induced peroxidation of lipid-esterified arachidonic acid and is considered a marker of systemic oxidative stress [1,2]. Additionally, the association between obesity and OS has also been observed in children and adolescents. In one study, the plasma levels of free malondialdehyde (MDA) and carbonyl groups were significantly higher in children with obesity (BMI ≥ 95%), while the activity of the antioxidant enzyme glutathione peroxidase (GPx) was higher and the concentration of its substrate, glutathione (GSH), was lower compared with non-obese children [3]. In another study, OS in children with obesity was evaluated indirectly by the plasma total antioxidant status (TAS), oxidative stress index (OSI), and total thiol level (TTL), which were significantly higher than in healthy controls [4]. In another TAS-related study, OS was indirectly assessed as being high in children with obesity by the low plasma total antioxidant capacity (TAC) and low levels of the natural antioxidants retinol, carotenes, and tocopherols found [5]. Furthermore, lower GSH and Cys-Gly levels and higher homocysteine concentrations and levels of oxidative stress markers, such as free thiobarbituric acid reactive substances (TBARS), 8-isoprostane, and protein carbonyl, have also been reported in children with obesity [6].

The present study aims to extend the evaluation of OS in children and adolescents with obesity using markers of OS stress involved in the early and late lipid oxidation process [7]. Aldehydic products of lipid peroxidation, such as free MDA and TBARS, being highly reactive, are better OS markers if evaluated bound to total proteins (Pr) and not as free [7]. Hence, the early and late OS-induced lipid peroxidation stages are represented correspondingly by the lipid peroxidation products lipid hydroperoxides (LOOH) and PrMDA/PrTBARS (other aldehydes except MDA). Specifically, the present study investigates the lipid peroxidation products LOOH, PrMDA, and PrTBARS in children and adolescents with obesity in order to be used as potential clinical diagnostic and/or prognostic markers of early-age obesity-associated OS-related diseases.

## 2. Materials and Methods

### 2.1. Subjects

The study included forty-one (41) children and adolescents with obesity, aged 5.75 to 16.33 years old (mean ± SD: 12.09 ± 2.93), and thirty-one (31) healthy age-matched control children and adolescents with normal BMI, aged 5.00 to 15.92 years old (mean ± SD: 10.74 ± 2.95). All were patients from the Department of Pediatric Endocrinology of the University Hospital of Patras in Greece, a teaching hospital that provides tertiary services for the geographical area of the Region of Western Greece (population coverage of ≈ 1,000,000). Exclusion criteria included a history of acute febrile illness during the previous two weeks, endocrinopathies, or any chronic disease that could affect the oxidative status of the participants. Children and adolescents who were externally referred to the outpatient clinic and subsequently proven to have had no substantial medical issues were used as controls. Obesity was defined as a BMI > 95% for age and sex, based on the Centers for Disease Control and Prevention (CDC) criteria, whereas a BMI of 10% to 85% was considered normal [8].

The population of children and adolescents with obesity consisted of 40% males and 60% females and none of them had been diagnosed with obesity-related comorbidities. Informed consent was obtained by all the participants and their parents after they were informed about the nature and purpose of the study. Participation in the study was voluntary. The study was conducted according to the guidelines of the Declaration of Helsinki and approved by the Institutional Review Board of the University General Hospital of Patras (Study Protocol No. 14633).

### 2.2. Lipid Peroxidation Markers

A marker of lipid hydroxyperoxide, LOOH, and its bound-to-oxidized-proteins aldehyde products, PrMDA and PrTBARS, were measured in the serum of fasting blood samples for the assessment of lipid peroxidation. To prevent artificial oxidation, 0.005 mL of 0.4 M butylated hydroxyanisole (BHA) was added to 2 mL of blood serum. In order to quantify LOOH, PrMDA, and PrTBARS, blood serum samples were subjected to a fractionation protocol used to isolate total serum proteins and lipids, as follows:

#### 2.2.1. Fractionation Protocol for Total Serum Lipid and Protein Isolation

1. 0.25 mL of blood serum is diluted with 0.5 mL ddH_2_O. To the diluted serum, 0.015 mL 1% DOC is added, followed by incubation for 10 min at room temperature. After incubation, 0.085 mL of 100% TCA is added and subsequent incubation in an ice-water bath is conducted for 20 min.
2. To the DOC-TCA treated sample, 0.025 mL of 3.4 M KCl and an equal volume (0.875 mL) of 2:1 (*v*/*v*) CHCl_3_: MetOH are sequentially added, followed by vortexing and centrifugation at 13,000× *g* for 5 min at 4 °C. This results in the formation of an upper aqueous phase, a middle solid protein disc, and a lower chloroform phase containing total lipids.
3. The chloroform phase is collected into a 2 mL microcentrifuge tube, and to the remaining aqueous phase and protein disc, 0.583 mL of CHCl_3_ is added, followed by vortexing and centrifugation at 13,000× *g* for 5 min at 4 °C. After centrifugation, the CHCl_3_ phase is combined with the initial CHCl_3_ phase and vacuum dried. The lipid fraction can be stored at −20 °C.
4. To the remaining aqueous phase and protein disc, 0.027 mL of 1% DOC and 0.175 mL of 100% TCA are added, followed by incubation for 10 min in an ice-water bath and centrifugation at 13,000× *g* for 5 min at 4 °C. The supernatant is discarded after centrifugation.
5. The remaining protein disc is 3×-washed with ice-cold acetone as described: 0.5 mL of acetone is added to the protein disc and with the narrow tip of a metallic spatula, the disc is dispersed, followed by centrifugation at 13,000× *g* for 5 min at 4 °C. The acetone supernatant is discarded. The 3× acetone-washed protein is vacuum-dried and stored at −20 °C.

#### 2.2.2. Total Protein Determination

To express each oxidative stress marker per total protein weight, the protein pellet is solubilized in the minimum volume of 50 mM NaOH and is quantified by a previously reported assay developed by our group [7].

#### 2.2.3. LOOH, PrMDA and PrTBARS Determination

LOOH and PrMDA were determined using an assay developed by our group [7] and subsequently modified for the measurement of PrTBARS in children and adolescents with type 1 diabetes mellitus [9]. Further details in the quantification of serum PrMDA, PrTBARS, and LOOH are presented in the supplementary data for lipid peroxidation markers.

### 2.3. Statistical Analysis

Statistical analysis was performed using SPSS v.24 (SPSS Inc., Chicago, IL, USA) and the significance level was set at 0.05. Continuous variables are presented as median values (minimum–maximum). The effect of independent variables on dependent variables was analyzed by the analysis of variance (ANOVA). ANOVA analyzes the difference between the means of more than two groups and was used to evaluate the influence of obesity on oxidative stress in a regression analysis. The non-parametric Mann–Whitney U test is used to compare differences between two independent groups when the dependent variable is either ordinal or continuous, but not normally distributed. The Mann–Whitney U test was used to explore the different impacts of obesity on oxidative stress markers according to sex. Pearson’s correlation was used to evaluate the relationship between quantitative variables.

Age-matching was performed using case–control matching with SPSS in order to reduce selection bias and improve internal validity.

## 3. Results

**Figure 1:**
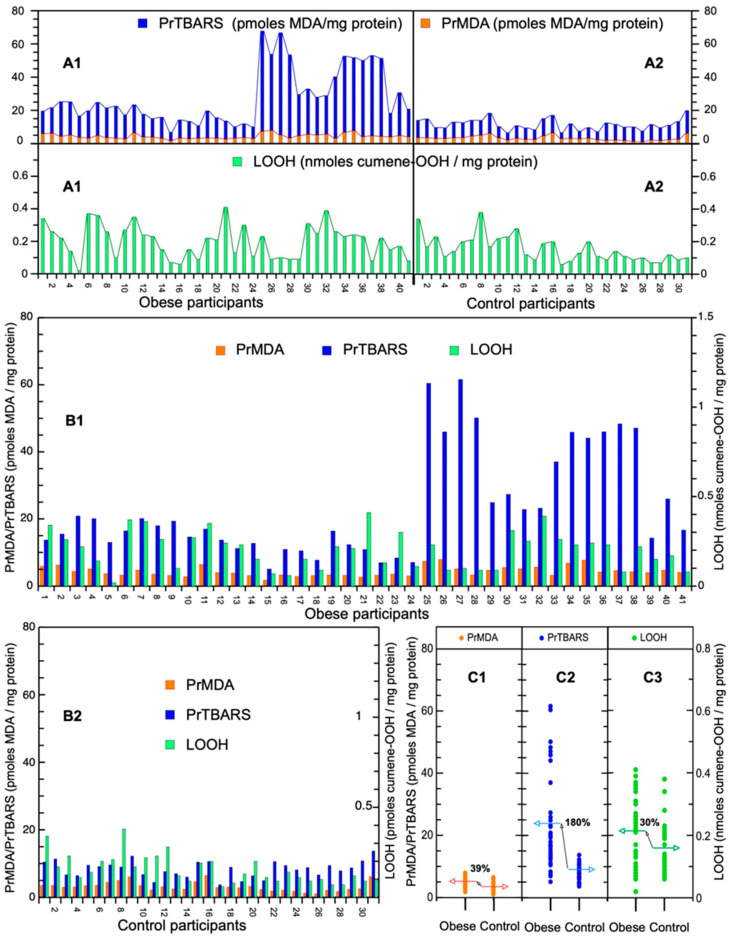
Serum PrMDA, PrTBARS, and LOOH concentrations of children and adolescents with obesity and controls. (**A**) Stacked column histogram of PrMDA and PrTBARS (in orange and blue, respectively), and histogram of LOOH (in green) in (**A1**) children and adolescents with obesity and (**A2**) controls. (**B**) Column histogram in column triplets of PrMDA, PrTBARS, and LOOH per each of (**B1**) the children and adolescents with obesity and (**B2**) the controls. (**C**) Scatter plots of (**C1**) PrMDA, (**C2**) PrTBARS, and (**C3**) LOOH for children and adolescents with obesity and controls. Horizontal arrows depict median values of the studied markers and their % difference between children and adolescents with obesity and controls is demonstrated.

The blood serum concentrations of LOOH, PrMDA, and PrTBARS of all the participants (including controls) are presented in Figure 1. Detailed marker values are listed in Table 1.

In Figure 1A1, a ~20-fold concentration difference between LOOH and its oxidative decomposition products PrMDA/PrTBARS is shown in the histogram with stacked PrMDA/PrTBARS separate from LOOH. Also, a ~4-fold-elevated concentration of PrTBARS over PrMDA is exhibited for both the obesity group and the controls. The variation in concentration levels among PrMDA, PrTBARS, and LOOH is displayed by the triplet column histograms for each of the children and adolescents with obesity (Figure 1B1) and the controls (Figure 1B2), emphasizing their metabolically personalized OS status. The distribution of the PrMDA, PrTBARS, and LOOH values are shown by the scatter plots in Figure 1C1–C3; their median value for the obesity group is increased by ~39%, ~180%, and 30%, respectively, in reference to the median line of the corresponding controls (0%). This increase is statistically significant for the PrMDA, PrTBARS, and LOOH markers (Table 2).

Of note, the PrTBARS levels are ~5.4-fold higher than PrMDA levels. Such comparison is valid because (i) both markers are expressed in the same units (pmoles MDA/mg protein), and (ii) both represent aldehydic lipid peroxidation fragments. LOOH molar levels (per mg protein) are ~22-fold and ~116-fold of PrMDA and PrTBARS molar levels, respectively (Figure 1). This comparison is also valid since PrMDA and PrTBARS originate mostly from the peroxidative decomposition of lipid hydroperoxides (LOOH).

Additional factors, such as sex and age, are also analyzed in reference to the levels of the PrMDA, PrTBARS, and LOOH markers. No statistically significant differences were found in the PrMDA, LOOH, and PrTBARS levels between males and females (Table 3). Moreover, the levels of the PrMDA, PrTBARS, and LOOH markers did not statistically change with age neither in children with obesity nor in the controls (Table 4).

## 4. Discussion

The present study demonstrates the quantification of the lipid peroxidation marker LOOH and its protein-bound decomposition aldehydic products, PrMDA and PrTBARS, by a TBA-based methodology that has been developed by our group [7,9].

LOOH, the early stage of lipid peroxidation, spontaneously decomposes into a complex mixture of highly reactive aldehydes (as well as ketones and alkanes), representing the late stage of lipid peroxidation [10,11], with their protein reaction products measured as PrTBARS in the present study. Among these many aldehydic products, MDA is considered the most highly toxic due to its dual aldehydic groups, which can readily oxidize proteins (measured as PrMDA in this study), and cause various modification types such as fragmentation, aggregation, and conformation changes, ultimately leading to alterations in the biochemical functioning of all tissue types [10,11]. Important biotoxic roles of MDA have already been identified in earlier research and are summed up elsewhere [12,13].

Our study shows a statistically significant increase in the concentrations of the OS markers PrMDA, PrTBARS, and LOOH (39%, 180%, and 30%, respectively) in children and adolescents with obesity, compared to controls (Figure 1C1). Previous studies have identified a TBARS marker that is not bound to proteins, in contrast to our PrTBARS marker, at increased levels in the serum of adult patients with type 2 Diabetes Mellitus (DM2) [14], and with diabetic neuropathy [15]. Similarly, and in contrast to our PrMDA marker, the MDA marker not bound to proteins has also been found at elevated concentrations in children with type 1 and type 2 Diabetes Mellitus (DM1 and DM2) and microalbuminuria [9,16]. We have also shown previously that PrTBARS and LOOH may serve as biomarkers of OS in children and adolescents with DM1, as well as potential prognostic clinical markers of DM1-related complications [9]. Both PrMDA and PrTBARS markers measure aldehydes of lipid peroxidation origin (with PrMDA specifically measuring the aldehyde MDA) and are expressed by the same concentration units (MDA/mg protein), therefore their molar sum is more representative of aldehydic lipid peroxidized protein-associated obesity. This is corroborated by our finding that the PrTBARS marker exceeds the PrMDA marker (Figure 1A1). This experimental outcome is expected as the PrTBARS marker represents the total aldehydic fragments resulting from the peroxidation of lipids, while the PrMDA marker represents only MDA, which, being a dialdehyde, is very reactive and thus more biotoxic. Moreover, the levels of PrTBARS and PrMDA, although at an average ratio of ~5.4-fold, do not show a constant proportionality between each other, neither among obese (and controls; Figure 1A2) nor when compared to the LOOH marker (also increased by 30% vs. controls), from the subsequent oxidation of which they mainly originate. This lack of proportionality among the PrMDA, PrTBARS, and LOOH markers may reflect a variable personalized OS status among patients, possibly because their OS defense is not completely compromised by obesity due to their young age. It should be noted that not all children and adolescents with obesity exhibited increased OS markers.

It can be concluded that PrTBARS levels appear to be mostly correlated with obesity in children among the three studied lipid peroxidation markers. This outcome is expected as the late lipid peroxidation product PrTBARS represents both an aldehyde collective and cumulative oxidation effect on proteins, compared to that of a single aldehyde, that of MDA alone, while the LOOH levels are non-cumulative, representing the early stage of lipid peroxidation.

Furthermore, knowing that there is a link between insulin resistance and oxidative stress [16,17,18,19], the two groups studied were age-matched. It is well-established that children normally experience transient insulin resistance at puberty [20]. Insulin resistance increases at the onset of puberty (Tanner stage 2) but returns to near prepubertal levels by the end of puberty (Tanner stage 5). Its peak occurs at Tanner stage 3 in both sexes [21,22], Therefore, in order to make a valid comparison, an age-matching process was followed.

The present study has some limitations including that the definition of obesity is based on the CDC criteria, according to which obesity is defined as a BMI above the 95th percentile for age and sex [8]. Although BMI represents the most widely accepted index for the evaluation of obesity in childhood, it should be acknowledged that it is not a direct measure of fat mass or adipose tissue distribution in the body, therefore it should be used with caution [23]. Alternative tools for the evaluation of body composition in childhood, such as fat mass (FM), fat mass index {FMI: fat mass (kg)/stature (m)^2^}, skeletal muscle mass index {SMI: skeletal muscle mass (kg)/stature (m)^2^}, skeletal muscle-to-body fat ratio (MFR) and DXA, were not considered as all of them have practical or other limitations, including the absence of a reference range adjusted for children and adolescents [24]. Bioelectrical impedance (BIA), a simple, portable, non-invasive method for assessing body composition, was also not used in the present study. Another limitation of the study is that parameters that could facilitate the interpretation of the results, such as the health status, nutritional habits, and lifestyle factors of the studied groups, were not assessed. Finally, although the differences described between the studied markers were statistically significant, generalizing the reported findings beyond the studied population should be performed with caution due to the relatively small sample used. Our findings require further exploration in larger populations.

In conclusion, the present study introduces the OS markers LOOH, PrTBARS, and PrMDA, with the latter two representing lipid peroxidation-caused protein oxidation damage, in the assessment of OS in children and adolescents with obesity. Further research is needed to clarify if these markers may also be useful as potential supplementary clinical diagnostic and/or prognostic markers of obesity and obesity-related complications in children and adolescents.

## Figures and Tables

**Table 1:** Oxidative stress marker values in children with obesity and controls.

Obesity/Control Samples	Obesity PrMDA	Control PrMDA	Obesity PrTBARS	Control PrTBARS	Obesity LOOH	Control LOOH
1	5.907	3.561	13.7	10.46	0.34	0.34
2	6.189	3.528	15.48	11.36	0.26	0.17
3	4.376	3.003	20.86	6.68	0.22	0.23
4	5.115	3.071	20.06	6.44	0.14	0.11
5	3.722	3.473	13.00	9.52	0.02	0.14
6	3.292	3.53	16.44	9.10	0.37	0.2
7	4.758	4.466	20.10	9.59	0.36	0.21
8	3.583	5.023	17.94	8.98	0.26	0.38
9	3.209	6.085	19.33	12.23	0.10	0.17
10	2.794	3.495	14.62	6.76	0.27	0.22
11	6.411	2.202	16.98	4.40	0.35	0.23
12	4.008	3.148	13.70	7.64	0.24	0.28
13	3.916	2.461	11.17	7.02	0.23	0.12
14	3.161	2.416	12.72	6.01	0.15	0.09
15	1.810	4.622	5.097	10.446	0.07	0.19
16	3.327	6.473	10.930	10.619	0.06	0.20
17	2.920	2.929	10.483	3.722	0.15	0.06
18	3.173	3.025	7.715	8.921	0.09	0.08
19	3.337	2.856	16.397	4.688	0.22	0.13
20	3.261	3.254	12.327	6.352	0.21	0.20
21	2.719	2.328	10.879	4.940	0.41	0.11
22	3.286	1.876	6.938	10.584	0.13	0.09
23	3.587	2.140	8.347	9.421	0.30	0.14
24	3.087	1.815	7.033	8.124	0.11	0.11
25	7.381	1.241	60.431	8.820	0.23	0.09
26	7.903	1.038	45.952	6.647	0.09	0.10
27	5.176	2.099	61.580	9.425	0.10	0.07
28	3.371	1.750	50.138	7.856	0.09	0.07
29	4.732	2.368	24.876	8.731	0.09	0.12
30	5.588	2.576	27.329	10.795	0.31	0.09
31	5.187	6.148	22.815	13.720	0.25	0.10
32	5.682		23.202		0.39	
33	3.272		37.000		0.26	
34	6.803		45.844		0.23	
35	7.686		44.107		0.24	
36	4.188		45.958		0.23	
37	4.692		48.337		0.08	
38	4.318		47.093		0.22	
39	4.029		14.306		0.15	
40	4.762		25.941		0.17	
41	4.111		16.659		0.08	

PrMDA and PrTBARS values are pmoles MDA/pg protein. LOOH values are presented in nmoles/cumene-OOH/pg protein.

**Table 2:** Oxidative stress markers in children and adolescents with obesity and controls.

	Children with Obesity (N = 41) Median (Min–Max)	Control (N = 31) Median (Min–Max)	*p*-Value
PrMDA (pmoles MDA/pg protein)	4.38 (1.81–7.90)	3.16 (1.04–6.47)	0.001
PrTBARS (pmoles MDA/pg protein)	23.51 (5.10–61.58)	8.38 (3.72–13.72)	0.001
LOOH (nmoles cumene-OOH/pg protein)	0.20 (0.02–0.41)	0.15 (0.06–0.38)	0.043

**Table 3:** Oxidative stress markers by sex in subjects with obesity and controls.

	Males with Obesity (N = 16)	Females with Obesity (N = 25)	*p*	Male Controls (N = 9)	Female Controls (N = 22)	*p*
PrMDA (pmoles MDA/pg protein)	4.66 (2.92–7.69)	4.21 (1.81–7.90)	0.283	3.43 (1.75–6.09)	3.05 (1.04–6.47)	0.356
PrTBARS (pmoles MDA/pg protein)	28.41 (7.72–61.58)	20.36 (5.10–60.43)	0.067	9.50 (6.01–12.23)	7.93 (3.72–13.72)	0.124
LOOH (nmoles cumene-OOH/pg protein)	0.23 (0.08–0.39)	0.19 (0.02–0.41)	0.320	0.15 (0.07–0.34)	0.16 (0.06–0.38)	0.716

Data are median with range in parentheses.

**Table 4:** Correlations between oxidative stress markers and age.

Group	PrMDA	PrTBARS	LOOH
Obese (N = 41)	−0.219 (0.181)	−0.207 (0.205)	0.081 (0.625)
Controls (N = 31)	0.031 (0.867)	0.261 (0.155)	−0.131 (0.483)

Dara are correlation coefficients r with *p* values in parentheses.

## Data Availability

The data presented in this study are available on request from the corresponding author. The data are not publicly available due to specific ethical and privacy considerations imposed by the Ethics Committee.

## Supplementary Materials

The following supporting information can be downloaded at: https://www.mdpi.com/article/10.3390/children11030314/s1. Supplement protocols for lipid peroxidation markers PrMDA, PrTBARS, LOOH.
