# Supplementary material for: New Clinical Markers of Oxidized Lipid-Associated Protein Damage in Children and Adolescents with Obesity
Source: Children (Basel). 2024 Mar 6;11(3):314. doi: 10.3390/children11030314 (PMC10969367; doi:10.3390/children11030314)
Supplement: Supplementary file 1 [file children-11-00314-s001.zip › children-2854181-supplementary.pdf]

Supplement protocols for lipid peroxidation markers PrMDA, PrTBARS, LOOH

Instrumentation

- Balance (Kern, Cat. No. 770/65/6J)
- Benchtop centrifuge (Hermle, Model Z206A)
- Double-beam spectrophotometer (Shimadzu, Model UV-1800)
- Microcentrifuge (Thermo Scientific, model Pico 17)
- Micropipettes (adjustable volume), 2.5 ml, 10 ml, 20 ml, 100 ml, 200 ml, and 1 ml, and tips (Eppendorf Research)
- Microcuvette for absorbance measurements, 12.5 x 45 mm external dimensions, 3 mm internal window, and 5 mm bottom (9/B/9/Q/10 quartz, 0.5 ml)
- Microcuvette for fluorescence measurements, quartz (Starna SOG/Q), 45 x 4 mm, 0.5 ml, with its FCA4 adapter
- Refrigerated microcentrifuge (Eppendorf Research, Model 5417R)
- Spectrofluorometer (Shimadzu, Model RF-1501)
- Thermoblock (FALC, model TD-150-P1)

Reagents

- Acetone (Sigma, cat. no. 34850)
- Ammonium iron (II) sulfate hexahydrate (FAS; Sigma, cat. no. 215406)
- Butanol-1 (ButOH; Merck, cat. no. 101990), caution, highly flammable
- Butylated hydroxyanisole (BHA; Sigma, cat. no. B1253)
- Chloroform ($CHCl_3$; Merck, cat. no. 1.02445), caution, highly flammable
- Cumene hydroperoxide (CumOOH; Sigma, cat. no. C0524), caution, oxidizing, corrosive
- Deoxycholic acid, sodium salt (DOC; Sigma, cat. no. D6750)
- Ethanol (EtOH; Merck, cat. no. 159010), caution, highly flammable
- Hydrochloric acid (HCl ≥37% w/w; Fluka, cat. no. 84415)
- Methanol (MetOH; Sigma-Aldrich, cat. no. 34860), caution, highly flammable
- Malonaldehyde bis(dimethyl acetal) (or 1,1,3,3-tetramethoxypropan) (MDA; Sigma, cat. no. 10,838-3)
- Potassium chloride (KCl; Merck, cat. no. 104933)
- Sodium hydroxide (NaOH; Merck, cat. no. 106462), caution, corrosive
- Sulfuric acid (H_2SO_4; Merck, cat. no. 100731), caution, corrosive
- 2-Thiobarbituric acid (TBA; Thermo scientific, cat. no. A12681.14)
- Trichloroacetic acid (TCA; Merck, cat. no. 100807), caution, corrosive
- Water (ddH_2O), purified by a Milli-Q system (Millipore Corp)
- Xylenol Orange, tetrasodium salt (XO; Alfa aesar, cat. no. 41379)

Reagent standard solutions

- 1 % w/v DOC: For 1 ml, dissolve 0.01 g DOC in 1 ml ddH_2O.
- 100 % w/v TCA: For 5 ml, dissolve 5 g TCA in 5 ml final volume ddH_2O.
- 3.4 M KCl: For 10 ml, dissolve 2.53 g KCl in 10 ml final volume ddH_2O.
- 2:1 (v/v) $CHCl_3$: MetOH: For 12 ml, mix 8 ml $CHCl_3$ with 4 ml MetOH.
- 50 mM NaOH: For 10 ml, dissolve 0.02 g NaOH in 10 ml ddH_2O.
- FOX-Fe reagent (2 mM XO, 0.25 M H_2SO_4): Dissolve 0.0076 g XO and add 0.07 ml H_2SO_4 in 4.5 ml ddH_2O, by stirring the solution for 30 min. Adjust volume to 5 ml. The reagent can be stored at -20°C.
- FOX+Fe reagent (2 mM XO, 5 mM FAS, 0.25 M H_2SO_4): Dissolve 0.0015 g FAS in 1 ml FOX-Fe reagent. The reagent must be prepared fresh before use.

- 5 mM CumOOH: dilute stock (5.52 M) to 5 mM with MetOH. The reagent is stable for a few hours at room temperature.
- 1 M NaOH: For 2 ml, dissolve 0.08 g NaOH in 2 ml ddH_2O.
- 0.2 M NaOH: For 2 ml, dissolve 0.016 g NaOH in 2 ml ddH_2O.
- TBA reagent: For 1 ml, mix equal volumes of the following solutions A and B.
  Solution A: Mix 0.625 ml 100 % TCA and 0.125 ml 37 % HCL for 0.75 ml final volume.
  Solution B: For 1 ml, dissolve 0.025 g TBA in 0.2 M NaOH.
- TBA solvent: For 1 ml, mix 0.5 ml solution A with 0.5 ml 0.2 M NaOH.
- 0.4 M BHA: For 2 ml, dissolve 0.144 g BHA in 2 ml EtOH.
- 0.1 M BHA: For 1 ml, mix 0.25 ml 0.4 M BHA with 0.75 ml EtOH.
- 2 μM MDA: dilute commercial stock (6 M) to 2 μM with 0.1 M NaOH. The reagent must be prepared fresh before use.

Blood serum samples
In 2 ml blood serum, 0.005 ml 0.4 M BHA is added to prevent artificial oxidation.

LOOH determination
Measurement of lipid hydroperoxides is performed to the isolated lipid fraction from blood serum by modification of a previously reported assay developed by our group, which quantifies simultaneously and specifically the main lipid peroxidation products PrMDA and LOOH (with sensitivity 20 and 100 pmol, respectively) (Grintzalis, K., Zisimopoulos, D., Grune, T., Weber, D., Georgiou, C. D. (2013). Method for the simultaneous determination of free/protein malondialdehyde and lipid/protein hydroperoxides. Free Radic. Biol. Med. 59:27-35). Lipids are solubilized in 0.1 ml MetOH and two sets of three different dilutions of sample (S) and sample blank (SB) are prepared by different quantities (x μl) of solubilized lipids in the same final volume, designed the first set as S_x and the second set as SB_x. Additionally, two sets of three reagent blanks are prepared, designed the first set as RB+Fe and the second set as RB–Fe. Each sample is prepared as described in the following Table and after 30 min incubation, the absorbance is measured at 560 nm. The net absorbance is calculated from the absorbance difference (S_x) – (RB+Fe) from which the absorbance difference (SB_x) – (RB–Fe) is subtracted. The net absorbance is converted to CumOOH concentration equivalents using the corresponding standard curve. For each sample, lipid hydroperoxide CumOOH equivalents are expressed as CumOOH nmole/mg protein.

S_x	S_{10}	S_{15}	S_{20}	SB_{10}	SB_{15}	SB_{20}	RB+Fe	RB–Fe
**Lipids in 0.1 ml MetOH (μl)**	10	15	20	10	15	20	-	-
**MetOH (μl)**	275	270	265	275	270	265	285	285
**FOX+Fe (μl)**	15	15	15	-	-	-	15	-
**FOX–Fe (μl)**	-	-	-	15	15	15	-	15

LOOH standard curve
To construct the LOOH standard curve, different CumOOH concentration reagent solutions at final volume 0.3 ml are prepared from the 5 mM stock, as shown in the following Table. Samples are incubated at room temperature for 30 min and centrifuged at 13,000 *g* for 5 min. Absorbance is measured at 560 nm.

CumOOH (mM)	0.25	0.5	1	1.5	2	2.5	3	3.5	4	0 (RB)
5 mM CumOOH (μl)	15	30	60	90	120	150	180	210	240	-
MetOH (μl)	270	255	225	195	165	135	105	75	45	285
FOX+Fe (μl)	15	15	15	15	15	15	15	15	15	15

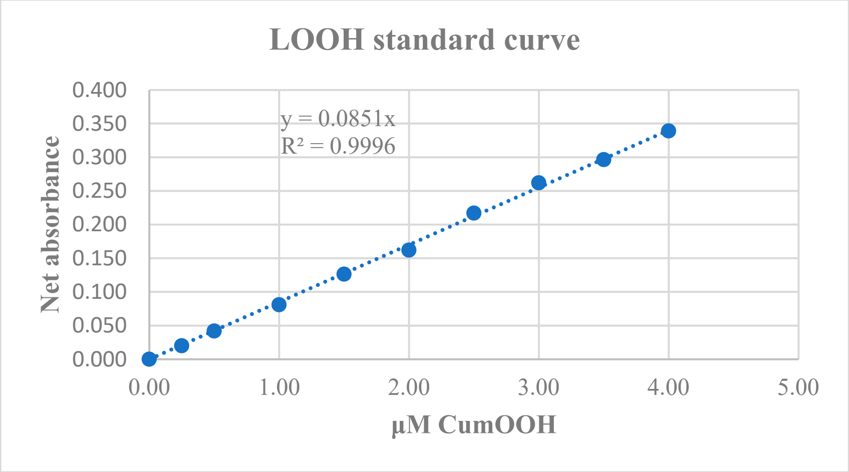

PrMDA / PrTBARS determination
Measurement of MDA bound to blood serum proteins is performed to the protein fraction which is initially solubilized in 50 mM NaOH for the protein determination assay and long-term storage of solubilized protein, by modification of a previously reported assay developed by our group. (Grintzalis, K., Zisimopoulos, D., Grune, T., Weber, D., Georgiou, C. D. (2013). Method for the simultaneous determination of free/protein malondialdehyde and lipid/protein hydroperoxides. Free Radic. Biol. Med. 59:27-35). Total proteins solubilized in 50 mM NaOH are adjusted to 0.1 M NaOH with 1 M NaOH and are incubated at 60°C for 30 min to enable hydrolysis of MDA from proteins. For the reaction protocol, two sets of two different dilutions of sample (S) and sample blank (SB) are prepared according to the following Table , by different quantities (x μl) of solubilized protein in the same final volume, designed the first set as S_x and the second set as SB_x. Additionally, three reagent blanks are prepared, designed as RB. For the reaction to take place, samples are incubated at 100°C for 20 min with the tube lids open. To extract the fluorescent reaction product an equal volume (0.3 ml) of ButOH is added to the samples followed by vortexing and centrifugation at 13,000 *g* for 5 min. The upper ButOH phase is isolated, and its fluorescent units (FU) are measured at ex/em 535/550 nm. Net FU is calculated from the FU difference (S_x) – (SB_x) – (RB) and is then converted to MDA pmoles using a corresponding standard curve of pure MDA. For each sample PrMDA is expressed as pmoles MDA/mg protein.
The ButOH phase is also measured for its absorbance at 535 nm (absorbance peak) and 600 nm (background) since TBA reacts also with other aldehydes than MDA, forming a colored product. Net absorbance is calculated from the absorbance difference ($S_{535} - S_{600}$) – ($SB_{535} - SB_{600}$) from which the absorbance difference ($RB_{535} - RB_{600}$) is subtracted. Net absorbance is then converted to MDA pmoles using a corresponding standard curve of pure MDA. For each sample PrTBARS are expressed as pmoles MDA/mg protein.

S_x	S_{80}	S_{160}	SB_{80}	SB_{160}	RB

**Protein solubilized in 0.1 M NaOH (µl)**	80	160	80	160	-
**0.1 M BHA (µl)**	3	3	3	3	3
**0.1 M NaOH (µl)**	167	87	167	87	247
**TBA reagent (µl)**	50	50	-	-	50
**TBA solvent (µl)**	-	-	50	50	-
Incubation at 100°C for 20 min. Extraction of the reaction product in 0.3 ml ButOH. Measurement of FU at ex/em 535/550 nm and absorbance at 535 nm, 600 nm.					

MDA, TBARS standard curves

To construct the MDA standard curve, different MDA concentration solutions at final volume 0.3 ml are prepared from the 2 µM stock, as shown in the following Table. Samples are incubated at 100°C for 20 min. After incubation, an equal volume of ButOH is added, followed by vortexing and centrifugation at 13,000 *g* for 5 min. The upper ButOH phase containing the reaction product is isolated and measured for its fluorescence and its absorbance.

**MDA (µM)**	**0.1**	**0.2**	**0.3**	**0.4**	**0.5**	**0.6**	**0.7**	**0.8**	**0.9**	**1**	**RB**
**2 µM MDA (µl)**	15	30	45	60	75	90	105	120	135	150	-
**0.1 M NaOH (µl)**	232	217	202	187	172	157	142	127	112	97	247
**TBA reagent (µl)**	50	50	50	50	50	50	50	50	50	50	50
**0.1 M BHA (µl)**	3	3	3	3	3	3	3	3	3	3	3

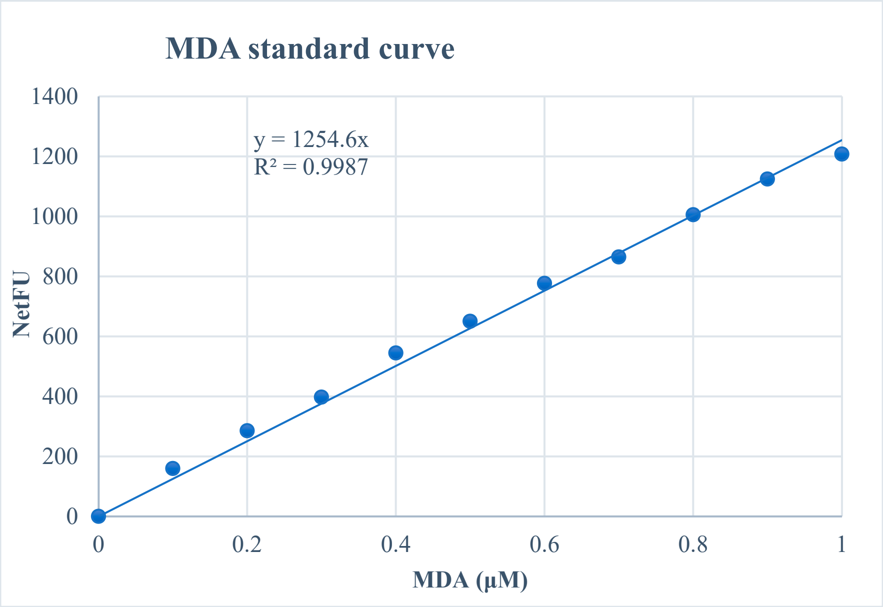
MDA standard curve
y = 1254.6x
R² = 0.9987
NetFU
1400
1200
1000
800
600
400
200
0
0
0.2
0.4
0.6
0.8
1
MDA (µM)

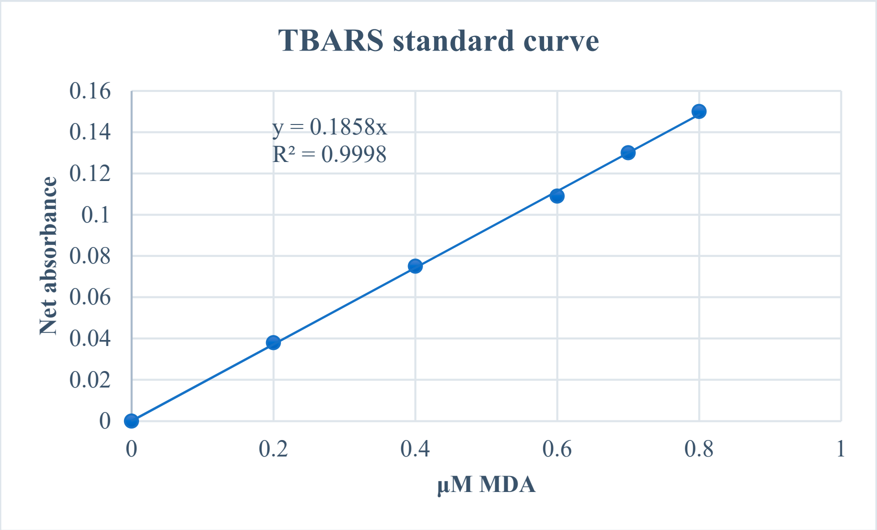
TBARS standard curve
y = 0.1858x
R² = 0.9998
Net absorbance
0.16
0.14
0.12
0.1
0.08
0.06
0.04
0.02
0
0
0.2
0.4
0.6
0.8
1
µM MDA
